# Detecting Spatial Patterns of Peatland Greenhouse Gas Sinks and Sources with Geospatial Environmental and Remote Sensing Data

**DOI:** 10.1007/s00267-024-01965-7

**Published:** 2024-04-02

**Authors:** Priscillia Christiani, Parvez Rana, Aleksi Räsänen, Timo P. Pitkänen, Anne Tolvanen

**Affiliations:** 1 Natural Resources Institute Finland (Luke), Oulu, Finland; 2 Natural Resources Institute Finland (Luke), Helsinki, Finland

**Keywords:** Greenhouse gases, Maximum entropy, Spatial distribution, Environmental Modelling, Peatland, Finland

## Abstract

Peatlands play a key role in the circulation of the main greenhouse gases (GHG) – methane (CH_4_), carbon dioxide (CO_2_), and nitrous oxide (N_2_O). Therefore, detecting the spatial pattern of GHG sinks and sources in peatlands is pivotal for guiding effective climate change mitigation in the land use sector. While geospatial environmental data, which provide detailed spatial information on ecosystems and land use, offer valuable insights into GHG sinks and sources, the potential of directly using remote sensing data from satellites remains largely unexplored. We predicted the spatial distribution of three major GHGs (CH_4_, CO_2_, and N_2_O) sinks and sources across Finland. Utilizing 143 field measurements, we compared the predictive capacity of three different data sets with MaxEnt machine-learning modeling: (1) geospatial environmental data including climate, topography and habitat variables, (2) remote sensing data (Sentinel-1 and Sentinel-2), and (3) a combination of both. The combined dataset yielded the highest accuracy with an average test area under the receiver operating characteristic curve (AUC) of 0.845 and AUC stability of 0.928. A slightly lower accuracy was achieved using only geospatial environmental data (test AUC 0.810, stability AUC 0.924). In contrast, using only remote sensing data resulted in reduced predictive accuracy (test AUC 0.763, stability AUC 0.927). Our results suggest that (1) reliable estimates of GHG sinks and sources cannot be produced with remote sensing data only and (2) integrating multiple data sources is recommended to achieve accurate and realistic predictions of GHG spatial patterns.

## Highlights

- We compared remote sensing and geospatial data to predict peatland greenhouse gas sinks and sources across Finland.
- Remote sensing data perform less effectively than habitat and climate-related variables.
- We recommend integrating various data sources for modeling greenhouse gas sinks and sources.

## Introduction

Greenhouse gas (GHG) emissions are a significant global concern due to their climate warming impact (IPCC 2022). Peatlands, particularly in northern landscapes, play a crucial role in the global carbon cycle, acting as substantial reservoirs of soil organic carbon (Harris et al. 2022). Despite covering only about 3% of the Earth’s terrestrial surface, these ecosystems store approximately 40% of the world’s soil organic carbon and hold between 10–15% of the global nitrogen pool (Hugelius et al. 2020; Leifeld and Menichetti 2018; Qiu et al. 2020; Treat et al. 2019).

In Finland, peatlands cover nearly one third of the country’s land area, totaling approximately nine million hectares. Of the peatlands, about half have been drained, mostly for forestry purposes (4.7 Mha) and to lesser extent to agriculture (0.3 Mha) and peat production (0.1 Mha) (Korhonen et al. 2021; Statistics Finland 2023). Typically, drained peatland soils serve as a source of CO_2_, whereas undrained peatlands act as sinks for CO_2_ and as sources for CH_4_ (Joosten and Clarke 2002; Kaat and Joosten 2009; Pönisch et al. 2023). While agricultural peatlands can be significant N_2_O sources (Anthony and Silver 2021; Ernfors et al. 2020; Leifeld and Menichetti 2018; Minasny et al. 2023), forested peatlands tend to be either minor sinks or sources for N_2_O (Leifeld 2018; Liu et al. 2020).

Given peatlands’ role in GHG dynamics, long-term and spatially extensive monitoring of GHG sinks and sources at regional and local levels is crucial for guiding climate change mitigation planning in the land use sector. Current conventional fieldwork methods such as chamber measurements (Holland et al. 1999; Lundegårdh 1927; Smith and Connen 2004; Zhao et al. 2023) and eddy covariance towers (Dou and Yang 2018; Foken et al. 2012), are labor-intensive, costly, and limited in their spatial coverage. Hence, there is an urgent need for economically viable methods to accurately measure GHG emissions across large spatial scales (Lees et al. 2018; Shono and Jonsson 2022; Wurtzebach et al. 2019).

Geospatial environmental data provides extensive coverage and can be used to estimate GHG sinks and sources. For example, Parkkari et al. (2017) showed that habitat conditions, such as drainage intensity and site fertility, which indirectly reflect moisture conditions, derived from geospatial data, were the most significant variables in explaining and predicting GHG balances at the landscape level. Webster et al. (2018) also found that climate i.e., mean diurnal range and seasonality of temperature, is an important driver in estimating peatland net emissions of CO_2_ and CH_4_. Furthermore, Koch et al. (2023) demonstrated the utility of machine learning techniques in modeling water table depth (WTD) on a national scale in Denmark using geospatial environmental data. Their study revealed that topography, water body proximity, and land use were crucial factors influencing WTD, which in turn is one of the most important factors affecting GHG emissions from peatlands (Abdalla et al. 2016; Huang et al. 2021). While geospatial data are invaluable for estimating GHG sinks and sources, they often suffer from coarse spatial resolutions, which limit their capacity to capture fine-scale landscape features and variations, and only offer a static snapshot of the landscape at a given moment.

Satellite-derived remote sensing data serves as a versatile tool for predicting GHG sinks and sources, offering global coverage, high temporal and spatial detail, and access to a wide variety of spectral regions to study GHG dynamics. Particularly beneficial is its capability to monitor peatlands that may be inaccessible due to wetness and open waters. For example, C-band synthetic aperture radar (SAR) Sentinel-1 can penetrate cloud cover, operate in darkness, and provide insights into surface vegetation structure and topography under various weather conditions (Bourgeau-Chavez et al. 2009; Karlson et al. 2019; Li et al. 2021; Millard et al. 2020; Räsänen et al. 2021; White et al. 2017). Additionally, SAR backscatter information is sensitive to soil moisture, a crucial factor influencing GHG fluxes in peatlands (Millard and Richardson 2018; Räsänen et al. 2022). Another valuable resource is multispectral optical remote sensing data from Sentinel-2, which enables monitoring of various physical and biological properties of peatlands (Lees et al. 2020; Räsänen et al. 2021,2022; Tucker et al. 2022), aiding in the detection of factors such as land cover, vegetation, water table depth, and soil moisture levels (Burdun et al. 2023; Räsänen et al. 2022), all of which significantly influence GHG emissions from peatlands (Abdalla et al. 2016; Lees et al. 2018). However, there are still relatively few studies that analyze the direct use of satellite data to predict GHG sinks and sources.

Some studies have highlighted the potential of remote sensing data in predicting the spatial patterns of GHGs. For instance, Räsänen et al. (2021) found that VH polarization data from Sentinel-1, along with water vapor, blue, and coastal aerosol bands from Sentinel-2, were important predictors for predicting CH_4_ fluxes in a heterogeneous peatland-forest-tundra landscape in northern Finland. Similarly, Junttila et al. (2021) identified strong relationships between CO_2_ gross primary productivity and a combination of Sentinel-2 Enhanced Vegetation Index 2 (EVI2), Sentinel-2-derived water scalar (W_s_), and daytime Land Surface Temperature (LST) from MODIS. However, to the best of our knowledge, there has been no attempt to detect spatial patterns of GHG sinks and sources at a national scale using directly remote sensing data.

This study builds upon the work of Parkkari et al. (2017), who utilized geospatial environmental data to detect peatland GHG sinks and sources. We expanded their approach by incorporating satellite remote sensing data (Sentinel-1 and Sentinel-2) as additional explanatory variables. Our research aimed to address the following questions: (1) How accurately can geospatial environmental and remote sensing data predict peatland GHG sinks and sources at a national scale? (2) How does remote sensing data compare with environmental data in terms of predictive accuracy? and (3) Do the predicted spatial patterns differ when using different explanatory variables?

## Materials and methods

### Study area

**Fig. 1:**
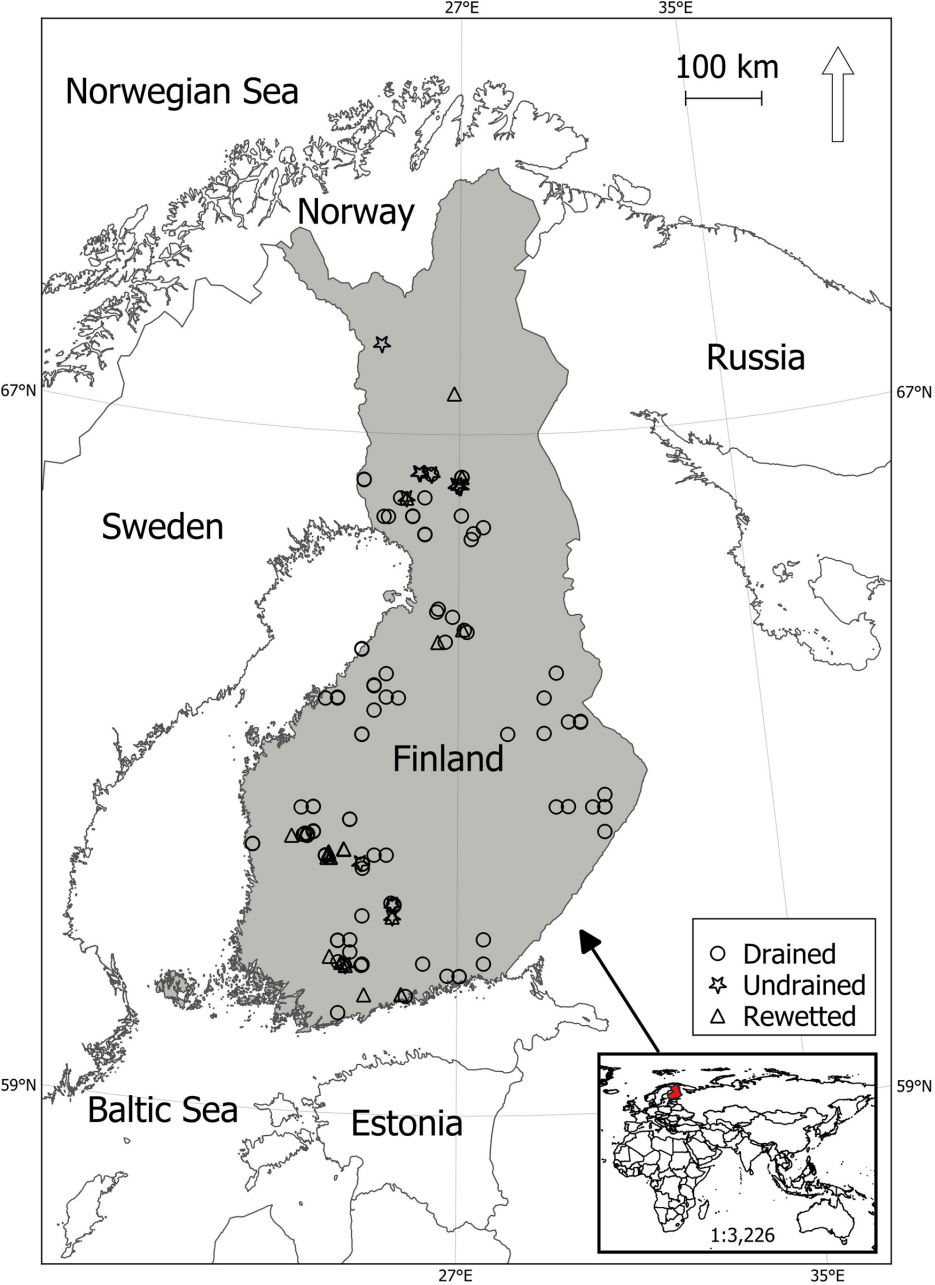
Location map of Finland, along with the distribution of GHG measurement sites

The study was carried out in Finland (60–70° N; 20–30° E) in Northern Europe (Fig. 1). The average annual temperature in the study area ranges from 6 °C in the southwestern region to −2 °C in the northeastern region, while annual precipitation varies between 500 mm and 750 mm in 1991–2020 (Jokinen et al. 2021).

We divided the study area into 1 ha grid cells (100 m × 100 m). We excluded the cells where peatlands covered less than 10% based on the peatland drainage status map from the Finnish Environmental Institute (2009). Consequently, a total of 13,382,854 spatial grid squares (133,829 km^2^) at a 1 ha resolution were used for predicting the distribution of GHG (44% of the total land area of Finland).

### Peatlands in Finland

The peatlands in Finland primarily consist of two main types: minerotrophic aapa mires, which are predominantly found in the middle and northern boreal vegetation zones, and ombrotrophic raised bogs, which are mainly located in the southern boreal zone (Ruuhijärvi 1983, 1988). Notably, drained peatland constitutes 41% of the peatland area in northern Finland, and 75% in southern Finland (Korhonen et al. 2021). There are five different main peatland fertility types for the forestry-drained peatlands based on their dominant ground vegetation and dominant tree species (Laine et al. 2018) (Table 1).

**Table 1:** Fertility types in forestry-drained peatlands and their characteristics

Peatland fertility types	Characteristics
1. Fertile herb-rich type (Rhtkg; *N* = 17)	Field layer vegetation is dominated by herbaceous plants while the dominant trees include Norway spruce (*Picea abies*), downy birch (*Betula pubescens*), and other deciduous trees.
2. Moderately fertile bilberry (*Vaccinium myrtillus*) type (Mtkg; *N* = 48)	Field layer is dominated by various shrubs and some herbaceous vegetation while the tree layer is a mix of Scots pine (*Pinus sylvestris*), Norway spruce, and downy birch.
3. The fertile lingonberry (*Vaccinium vitis-idaea*) type (Ptkg; *N* = 35)	Shrub-dominated field layer and pine-dominated tree layer with some downy birch and Norway spruce.
4. The nutrient-poor shrub type (Vatkg; *N* = 27)	Abundant occurrence of dwarf-shrubs typically associated with pine bogs. Moss layer consists of various mosses, including Sphagnum sp. Tree layer is mostly composed of Scots pine.
5. Lichen type (Jätkg; *N* = 16)	The diversity of vascular plants is generally low compared to other types of drained peatlands. Ground layer is dominated by mosses (especially *S. fuscum*) and lichens. Tree layer is mostly composed of Scots pine.

*N* = number of sites

These types also align with undrained peatlands, which, however, are dominated by mire vegetation such as *Carex* spp, *Sphagnum* spp, and selected forbs and shrubs. Undrained peatlands can have tree cover, but wetter undrained peatlands lack it.

### Field-based GHG sinks and sources data

**Fig. 2:**
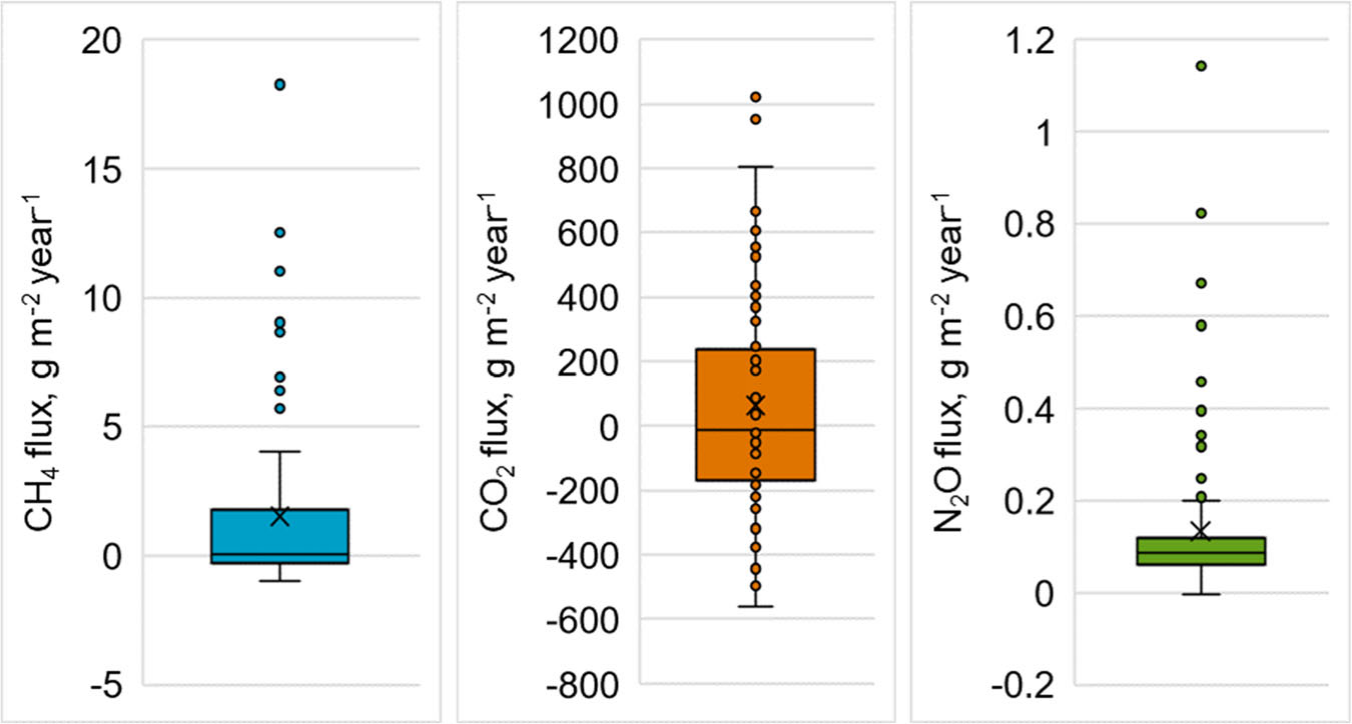
Boxplot visualizing field-based greenhouse gas sinks and sources data. The box displays the interquartile range (IQR) with the median depicted as the middle line. The ‘x’ represents the mean, and the whiskers extend to the minimum and maximum values, providing a visual representation of the data distribution. Negative values on the *y*-axis indicate GHG uptake from the atmosphere, while positive values signify GHG emissions into the atmosphere

We used field-based data on soil-atmosphere fluxes of CH_4_ (79 drained, 21 rewetted, and 3 undrained sites), CO_2_ (including heterotrophic and total soil respiration, measured in 76 drained sites), and N_2_O (59 drained, 24 rewetted, and 20 undrained sites) (Table 2, Fig. 2). From the temporal, GHG measurements, that were conducted predominantly during the snow and frost-free seasons, annual soil balances of CH_4_, N_2_O, and CO_2_ were derived. The detailed methodology for estimating these annual balances can be found in the referenced papers (Korkiakoski et al. 2019; Minkkinen et al. 2018, 2020; Ojanen et al. 2010, 2013, 2018, 2019).

**Table 2:** List of various studies contributing GHG flux data used in this study

Source	Site	Method
Ojanen et al. 2010, 2013	68 study sites from permanent sample plots of the 8th NFI	For CO_2_ flux, both total soil respiration (R_TOT_) and heterotrophic respiration (g m − 2 h − 1 of CO_2_) were measured from 5 points at each study site. Respiration was measured every 2–3 weeks using a portable infrared gas analyzer with an opaque closed chamber from May to October 2007 and 2008. Gas samples for calculating CH_4_ and N_2_O fluxes were taken from 4 of the R_TOT_ points 5–7 times. Samples were collected from the chamber headspace using syringes at 5, 15, 25, and 35 min after inserting the chamber into the point.
Minkkinen et al., 2018	Kalevansuo (was drained in 1971)	The net ecosystem exchange of CO_2_ was measured using the eddy covariance method from a mast positioned above the forest canopy from April 2005 to April 2006. Additionally, CO_2_ fluxes from the soil and forest floor were assessed using closed chambers, with a focus on four plots, each with 16 measurement points, between 2005 and 2008. Soil CH_4_ fluxes were measured with static chambers, and fluxes were measured from four points on two parallel ditches on both sides of the mast, conducted a total of 7 times between June and December 2011.
Ojanen et al. 2019	Six study sites in Finland which were identified as low-productivity drained peatlands, categorized as either nitrogen-rich or nitrogen-poor areas. These sites had undergone long-term fertilization experiments with varying fertilizer types and doses, conducted by the Natural Resources Institute Finland.	At each plot, six measurement points were established. Gas fluxes were measured a total of 11 times at each plot during the snow-free period between July 2014 and September 2015. Respiration measurements were conducted using a portable infrared gas analyzer with an opaque closed chamber. Additionally, gas samples for calculating CH_4_ and N_2_O fluxes were collected from the chamber headspace at 5, 10, 15, and 20 min after placing the chamber at the measurement point.
Korkiakoski et al. 2019	Lettosuo (was drained manually in the 1930s and more effectively in 1969)	Eddy covariance was utilized to measure CO_2_ fluxes from April 2016 to March 2018, while chamber methods were employed to measure CO_2_, CH_4_, and N_2_O fluxes from June 2015 to August 2017, respectively. Chamber measurements were mostly conducted during snow-free periods, with intervals ranging from one week to one month.
Minkkinen et al. 2020	28 undrained, 65 forestry-drained (6 of which were fertilized experimental sites), and 24 rewetted boreal peatland study sites in Finland.	Flux measurements were carried out using the closed chamber method, with gas samples collected into four syringes at equal intervals of either 5 or 10 min (with incubation times of 20 or 35 min). Measurements were conducted primarily during the snow-free season (May–October) but occasionally during winter as well.

Using the annual GHG balance data, we calculated the measurement sites as either sources (emitting GHGs into the atmosphere) or sinks (absorbing GHGs from the atmosphere) for each GHG. Sites with a balance value of 0 were designated as sinks. The distribution of measurement sites categorized as sinks or sources for each GHG was as follows: 47 sinks and 56 sources for CH_4_, 39 sinks and 37 sources for CO_2_, and 1 sink and 102 sources for N_2_O (Table 3).

**Table 3:** Numbers of measurement sites of field based GHG sinks and sources data

GHG	Drained		Undrained		Rewetted		Total
	Sink	Source	Sink	Source	Sink	Source	
CH_4_	47	32	-	3	-	21	103
CO_2_	39	37	-	-	-	-	76
N_2_O	-	59	1	19	-	24	103

### Geospatial environmental data

In selecting our geospatial environmental variables, we drew guidance from the findings of Parkkari et al. (2017), who emphasized the significance of drainage and moisture-related variables in predicting GHG sinks and sources. For our study, we derived ten explanatory geospatial environmental variables and grouped them into three categories: climate, topography, and habitats (Table 4). All the variables were then resampled into 100 m spatial resolution using the nearest neighbor method. To ensure there was no multicollinearity among the variables, we applied Spearman’s rank correlation, setting a pairwise absolute correlation cutoff at 0.70, as recommended by McCune (2016).

**Table 4:** List and description of geospatial environmental variables used in the study.

Geospatial environmental variables	Abbreviation	Unit	Resolution	Data source	Mean [min-max]
Climate variables					
Growing degree days	GDD	–	1 × 1 km	FMI	1112.28 [705.94–1418.10]
Mean water balance	WAB	mm/year	1 × 1 km	FMI	306.36 [144.70–383.19]
Topography variable					
Mean topographic wetness index	TWI	–	16 × 16 m	NLS, DEM	7.44 [5–12]
Habitat variables					
Proportion of drained peatlands in grid square of peatland area	DRAINED	%	25 × 25 m	SYKE	37.15 [0–98.70]
Proportion of undrained peatlands in grid square of peatland area	UNDRAINED	%	25 × 25 m	SYKE	10.26 [0–82.95]
Mean root biomass (spruce, pine, other broadleaves)	ROOT_BIOMASS	10 kg/ha	16 × 16 m	Luke, MS-NFI	412.71 [0–1201.65]
Proportion of Herb-rich type in grid cell area	Rhtkg	%	16 × 16 m	Luke, MS-NFI	0.6 [0–8.81]
Proportion of Vaccinium myrtillus types I and II in grid cell area	Mtkg	%	16 × 16 m	Luke, MS-NFI	5.85 [0–37.28]
Proportion of Vaccinium vitis-idaea types I and II types in grid cell area	Ptkg	%	16 × 16 m	Luke, MS-NFI	35.82 [0–83.52]
Proportion of Cladina type in grid cell area	Jätkg	%	16 × 16 m	Luke, MS-NFI	2.70 [0–43.37]

*FMI* Finnish Meteorological Institute, *NLS* National Land Survey, *DEM* Digital Elevation Model, *SYKE* Finnish Environment Institute, *Luke* Natural Resources Institute Finland, MS-NFI Multi-source National Forest Inventory

For the climate category, we calculated mean growing degree days (GDD) and mean water balance (WAB) annually using climate data from the Finnish Meteorological Institute spanning the years 1990–2013 (Pirinen et al. 2012). GDD, determined by daily mean temperatures, acts as an indicator of plant growth development, considering both the duration of the growing season and solar energy influx (Skov and Svenning 2004). Recognizing that precipitation alone does not fully represent available moisture for plants, we calculated the water balance by subtracting monthly potential evapotranspiration from precipitation, with monthly balances then aggregated annually. These climatic variables influence organic matter decomposition rates, the balance between plant photosynthesis and respiration, and water availability within peatlands, all factors affecting GHG fluxes in these ecosystems (Antala et al. 2022; Górecki et al. 2021).

In the topography category, we included the topographic wetness index (TWI; Beven and Kirkby 1979), calculated from a 2 m spatial resolution digital terrain model using local slope and upslope contributing area (Salmivaara 2016). Topography plays a crucial role in water flow and accumulation across landscapes, influencing nutrient availability and plant productivity, thereby indirectly affecting GHG fluxes (Murphy et al. 2009; Stewart et al. 2014).

In the habitat category, we used seven variables. Five of these variables were derived from multi-source national forest inventory (MS-NFI) data from 2017 (Natural Resources Institute Finland 2017), including the proportion of tree species root biomass and the proportion of four site fertility types (Rhtkg, Mtkg, Ptkg, Jätkg). Additionally, we calculated the proportion of undrained and drained peatlands in each grid cell using the peatland drainage status dataset provided by the Finnish Environment Institute (2009), which was based on the topographic database of the Finnish National Land Survey.

### Remote sensing data

The remote sensing dataset comprised European Space Agency (ESA) Copernicus Sentinel-1 and Sentinel-2 data, acquired from Google Earth Engine (GEE; Gorelick et al. 2017). To filter out noise that exists in individual images, we calculated representative imagery for three specific periods within the snow and frost-free season: early summer (ES, May 1 - June 15), mid-summer (MS, July 1 - August 15), and late summer (LS, September 1 - October 15) from 2019 to 2023 (Table 5). These periods correspond to different stages of the growing season: ES represents high-water table conditions after snowmelt at the beginning of the growing season, MS indicates a period of limited water supply and peak vegetation growing season, and LS represents end of the growing season when vegetation senesces and when water table is again higher. We calculated multi-year averages since multi-year data is more representative for the average conditions of the different periods and corresponds better with the field data which is also based on multi-year averages. For Sentinel-2, we included only ES and MS due to persistent cloud-cover in Finland during LS. Sentinel-1 has a 20 m resolution, while Sentinel-2, as well as the derived indices, have a resolution of 10 m.

**Table 5:** List and description of remote sensing variables used in the study

Variable	Abbreviation	Band(s)	Equation
Sentinel 1			
Vertical transmit vertical receive	ES_VV MS_VV LS_VV		
Vertical transmit horizontal receive	ES_VH MS_VH LS_VH		
Polarization ratio (VV/VH)	ES_POL MS_POL LS_POL		
Sentinel 2			
Individual bands:			
Band 2	ES_BLUE	Blue	
	MS_BLUE	Blue	
Band 3	ES_GREEN	Green	
	MS_GREEN	Green	
Band 4	ES_RED	Red	
	MS_RED	Red	
Band 5	ES_RE1	Red edge	
	MS_RE1	Red edge	
Band 6	ES_RE2	Red edge	
	MS_RE2	Red edge	
Band 7	ES_RE3	Red edge	
	MS_RE3	Red edge	
Band 8	ES_NIR	NIR	
	MS_NIR	NIR	
Band 11	ES_SWIR1	SWIR	
	MS_SWIR1	SWIR	
Band 12	ES_SWIR2	SWIR	
	MS_SWIR2	SWIR	
Indices:			
Modified normalized difference water index	ES_MNDWI MS_MNDWI	Green, SWIR1	$\frac{({Green}-{SWIR})}{({Green}+{SWIR})}$
Normalized difference moisture index	ES_NDMI MS_NDMI	NIR, SWIR1	$\frac{({NIR}{-}{SWIR})}{({NIR}+{SWIR})}$
Normalized difference vegetation index	ES_NDVI MS_NDVI	Red, NIR	$\frac{({NIR}-{RED})}{({NIR}+{RED})}$
Normalized difference water index	ES_NDWI MS_NDWI	Green, NIR	$\frac{({Green}{-}{NIR})}{({Green}+{NIR})}$

ES, MS, and LS refer to early, mid and late summer data acquisition periods, respectively

For Sentinel-1 data, we used the Ground Range Detected, Sentinel-1 Toolbox preprocessed data that had undergone thermal noise removal, radiometric calibration, and terrain correction with ASTER DEM. We used only ascending orbit imagery for our analysis and calculated the median for different time periods. Alongside the vertical transmit vertical receive (VV) and vertical transmit horizontal receive (VH) polarization bands, we calculated their ratio (referred to hereafter as the Polarization ratio) and included it in the explanatory variables for remote sensing data.

We utilized Sentinel-2 Level-2A (atmospherically corrected surface reflectance) images with a maximum cloud cover of 20%. We masked out remaining clouds, cloud shadows, and snow with Scene Land Cover pixel classification. We used the unmasked image areas to generate a mosaic, with each pixel representing band-wise 40^th^ percentile reflectance values. Compared to the more conventional median-based approach (Kollert et al. 2021; Shafeian et al. 2021), this method yielded a better outcome with fewer cloud remnants and haze, while still effectively avoiding low-reflectance areas caused by cloud shadows (Pitkänen et al. 2024). We utilized nine bands, excluding those with 60 m initial resolution (bands 1, 9, and 10) and the narrow near-infrared band (8A). Additionally, we calculated four spectral indices, including the Modified Normalized Difference Water Index (MNDWI; Xu 2006), Normalized Difference Moisture Index (NDMI; Gao 1996), Normalized Difference Vegetation Index (NDVI; Rouse et al. 1974), and Normalized Difference Water Index (NDWI; McFeeters 1996). Finally, to reduce the computation time of the analyses and to match with the spatial resolution of geospatial environmental variables, we resampled the Sentinel-1 and Sentinel-2 to a 100 m pixel resolution using the nearest neighbor method.

### GHG model calibration and validation

We utilized the maximum entropy (MaxEnt), a machine-learning algorithm to predict the spatial patterns of GHG sinks and sources. The core principle of the MaxEnt is to achieve the highest possible entropy in the distribution (Phillips et al. 2006), resulting in a probability distribution model that connects explanatory variables with occurrence records (Elith et al. 2011; Merow et al. 2013; Phillips et al. 2006; Phillips and Dudík 2008). We chose this method because it efficiently handles complex predictor interactions and non-linearity, and is suitable for dealing with small sample sizes (Parkkari et al. 2017; Phillips et al. 2017; Saarimaa et al. 2019). Although MaxEnt is traditionally used in species distribution modeling, it has also been successfully applied in modeling GHG sinks and sources (Parkkari et al. 2017). Following the approach by Parkkari et al. (2017), we employed the default parameter settings, including a regularization multiplier of 1, auto-features, a maximum of 500 iterations, and a convergence threshold of 10^−5^.

We treated the measured GHG sink or source information as presence-occurrence data and compared it against 10,000 randomly selected background points representing the distribution of environmental conditions and remote sensing features in the study area. We calculated the mean values of the environmental and remote sensing variables within a 50-meter radius circular buffer area surrounding each GHG measurement point and background point. Employing the buffer area helps eliminate potential noise in individual pixels, thereby avoiding the issue of misleading values when points are located near the edge of pixels.

We developed individual models for CO_2_, CH_4_, and N_2_O sinks and sources. However, we did not include the N_2_O sink in our analysis since data only from one site was available. We constructed separate models for the following explanatory variable sets (1) geospatial environmental data, (2) remote sensing data, and (3) a combination of both types of data.

To evaluate our model, we employed a 10-fold cross-validation and reported the average results over all iterations. We used the area under the receiver operating characteristic curve (AUC) to assess the model performance. The AUC is a widely recognized, effective, and threshold-independent metric for evaluating distribution modeling (Rana and Tolvanen 2021; Saarimaa et al. 2019; Zhang et al. 2018). Model accuracy was considered low if AUC fell below 0.7, fair if it ranged from 0.7 to 0.8, good if between 0.8 and 0.9, and excellent if the AUC exceeded 0.9 (Saarimaa et al. 2019; Swets 1988). We evaluated model stability by comparing the test AUCs to the training AUCs (Parviainen et al. 2013):

$${AUC}\,{stability}=\frac{{Test}\,{AUC}}{{Training}\,{AUC}}$$

A closer similarity between the test and training AUCs indicates greater model stability.

We utilized MaxEnt permutation importance analysis to identify key variables for our models. This approach was chosen for its robustness, as permutation importance relies solely on the final MaxEnt model, regardless of the path taken to achieve it. By randomly permuting variable values among training points—both presence and background—and assessing the resulting decrease in training AUC, we estimated each variable’s contribution. A substantial decrease indicates a variable’s significant impact on the model. Therefore, MaxEnt permutation importance emerges as a superior metric for evaluating a variable’s explanatory power due to its independence from the specific algorithmic path taken (Saarimaa et al. 2019).

Given the recommendation to use only relevant variables in the modeling process (Elith et al. 2011; Parkkari et al. 2017), we initially ran the model using all variables and assessed the permutation importance values. Subsequently, we iteratively removed the variable with the lowest importance value, following a backward stepwise procedure, until there was no further increase in model performance. This approach aimed to strike a balance between retaining potentially important variables and preventing the inclusion of irrelevant ones. Finally, we selected the variable combination with the highest test AUC as the final model. Finally, we generated GHG sink and source prediction maps for the study area to visualize the spatial distribution of GHGs.

## Results

**Fig. 3:**
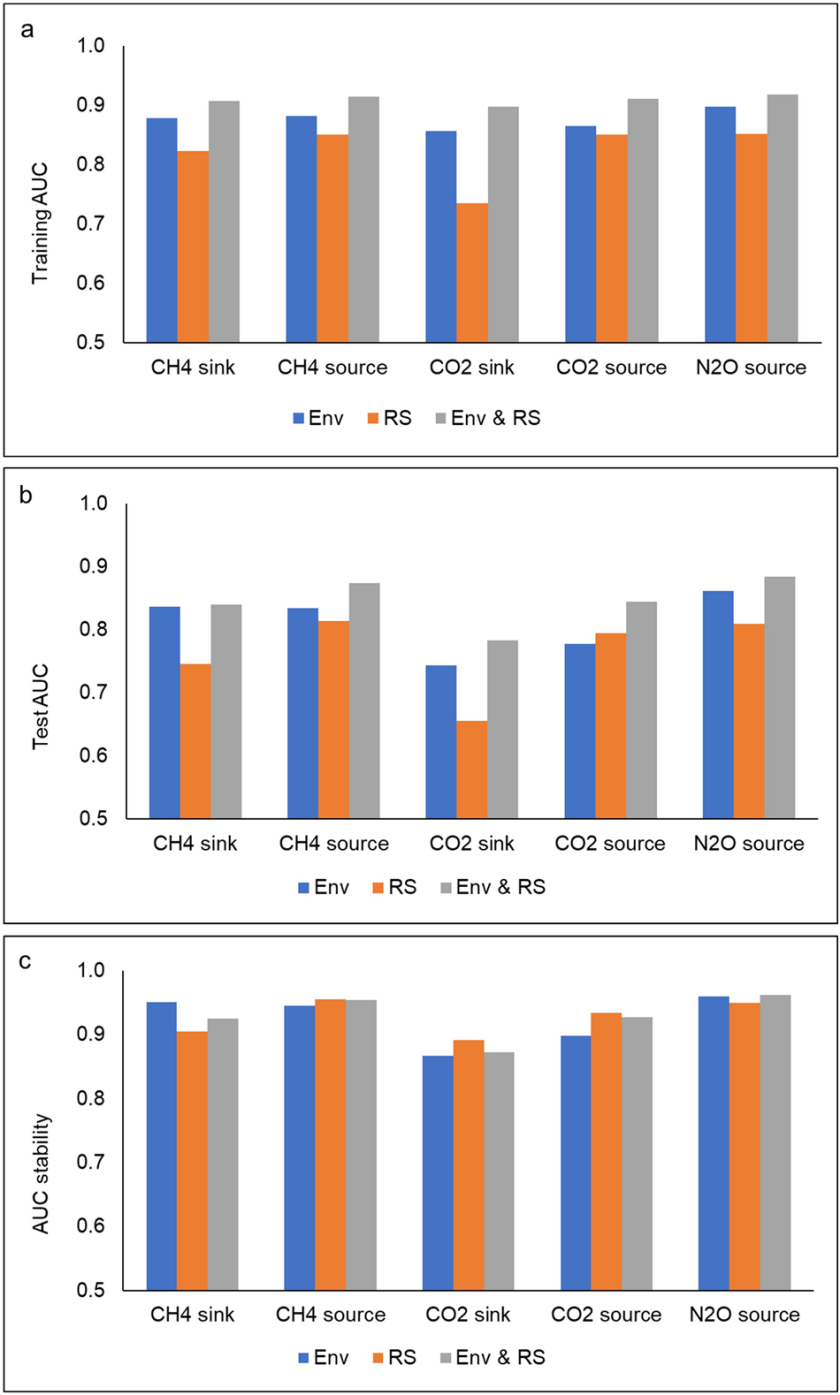
Training (**a**) and test (**b**) AUC values, and AUC stability (**c**) of the models using geospatial environmental (Env) and remote sensing (RS) variables

The model that incorporated both geospatial environmental and remote sensing variables yielded the highest AUCs, with 0.845 for the test and 0.910 for the training data (Fig. 3a, b). Models that incorporated environmental variables performed almost as well, yielding average AUCs of 0.810 and 0.876 for the test and training sets, respectively. However, models based on remote sensing variable lagged behind, with average AUCs of 0.763 and 0.823 for the test and training data, respectively. The models from remote sensing variables only were slightly more stable (AUC stability of 0.927) than those from geospatial environmental variables (AUC stability of 0.924), while the combination of both types of variables exhibited the highest stability (AUC stability of 0.928) (Fig. 3c).

In the variable importance analysis for models utilizing only geospatial environmental variables, GDD emerged as the most influential environmental variable, being the most important variable for CH_4_ sinks and N_2_O sources and having high importance in other models (Table 6). For CH_4_ and CO_2_ sources, DRAINED and UNDRAINED were the most important variables, respectively, and mtkg was the most important for CO_2_ sinks. Notably, GDD, UNDRAINED, DRAINED, WAB, and peatland fertility types ranked among the top three most important variables for all GHGs, surpassing ROOT_BIOMASS and TWI.

**Table 6:** Top three geospatial environmental (Env) or remote sensing (RS) variables in the final models of each GHG based on their permutation importance values

Model	Env	Permutation	RS	Permutation		Env & RS	Permutation
CH_4_ sink	GDD	35.395	ES_Blue		18.793	UNDRAINED	23.162
	UNDRAINED	27.646	MS_RE1		18.753	GDD	17.707
	jatkg	15.761	MS_NDWI		16.461	MS_NDWI	14.872
CH_4_ source	DRAINED	30.556	MS_NDMI		17.920	ES_MNDWI	13.368
	GDD	18.838	ES_RED		13.772	GDD	11.681
	WAB	17.154	MS_NDVI		13.458	MS_NDVI	11.056
CO_2_ sink	mtkg	19.095	LS_VH		63.547	UNDRAINED	15.372
	UNDRAINED	17.389	MS_Blue		15.087	GDD	12.331
	ptkg	17.094	ES_Blue		9.486	DRAINED	10.945
CO_2_ source	UNDRAINED	35.425	MS_RE1		33.509	MS_RE1	18.083
	DRAINED	27.309	ES_NIR		18.890	UNDRAINED	17.146
	GDD	14.747	ES_RE2		15.542	GDD	14.142
N_2_O source	GDD	28.753	ES_MNDWI		17.812	WAB	17.017
	WAB	27.255	ES_RED		11.752	GDD	16.481
	DRAINED	19.787	MS_MNDWI		8.448	ES_NIR	15.762

When considering remote sensing variables, Sentinel-2 variables predominated in the models, except for CO_2_ sinks, for which Sentinel-1 LS_VH was the most important. In more specific, individual bands ES_Blue and MS_RE1, emerged as the most influential for models predicting CH_4_ sinks and CO_2_ sources, respectively, while spectral indices MS_NDMI and ES_MNDWI were deemed the most important in predicting CH_4_ and N_2_O sources, respectively (Table 6).

When utilizing both geospatial environmental and remote sensing variables, UNDRAINED was the most influential variable for CH_4_ and CO_2_ sinks, WAB for the N_2_O sources, and ES_MNDWI and MS_RE1 for CH_4_ and CO_2_ sources, respectively (Table 6). Interestingly, the top three most important variables were a mix of geospatial environmental and remote sensing variables, except for CO_2_ sinks, for which all top three variables were environmental geospatial ones.

**Fig. 4:**
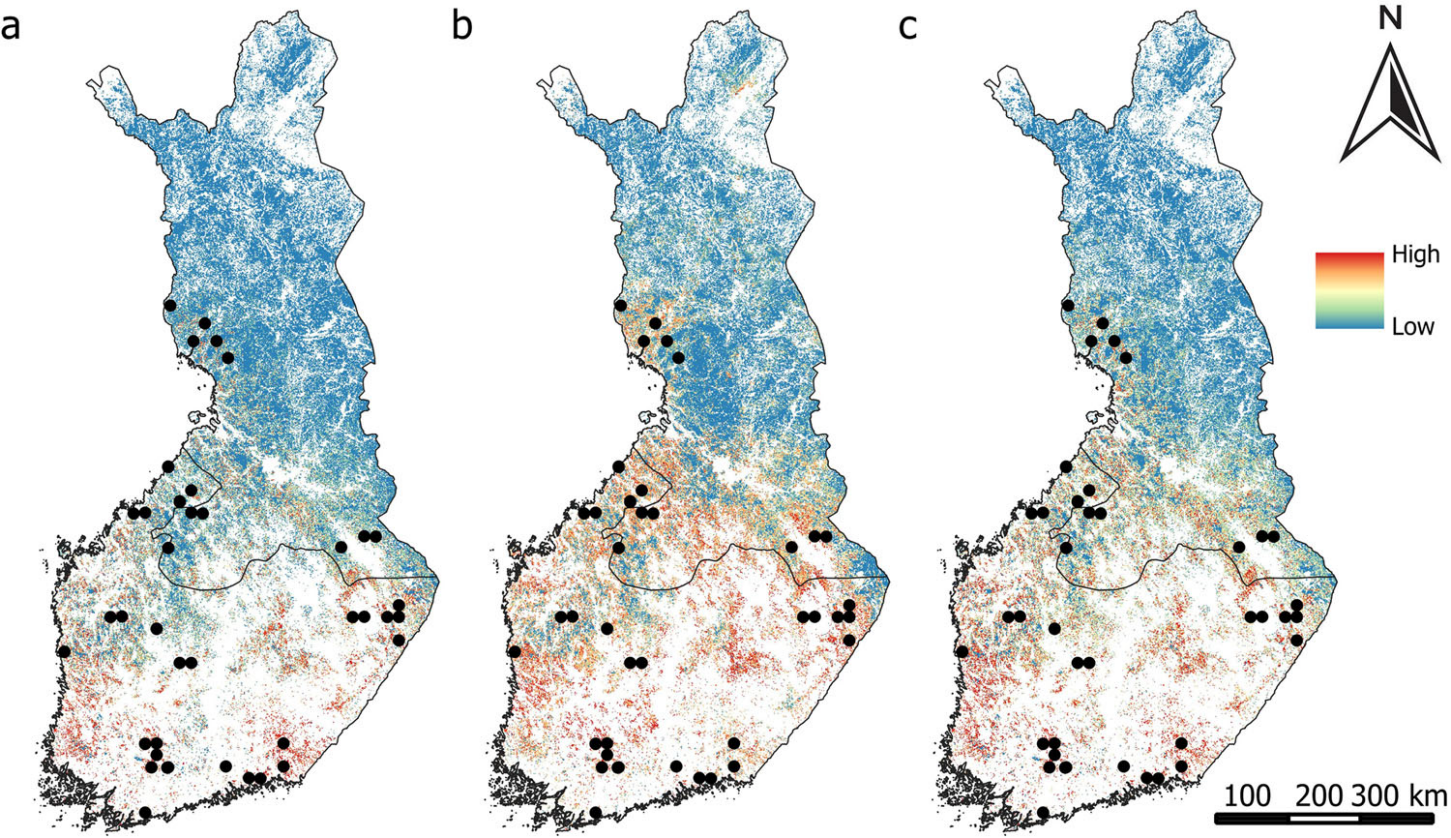
Predicted probability of CH_4_ sinks from (**a**) geospatial environmental variables, (**b**) remote sensing variables, (**c**) both geospatial environmental and remote sensing variables. Black dots represent the measurement sites of CH_4_ sinks

Figure 4a–c exhibited a similar distribution pattern for CH_4_ sinks. All variable sets predicted CH_4_ sinks in Finland’s central to southern region, with smaller occurrences observed in the northwestern part. Maps generated from remote sensing variables depicted a higher probability of CH_4_ sinks, evident by the presence of more red colors on the map (Fig. 4b).

**Fig. 5:**
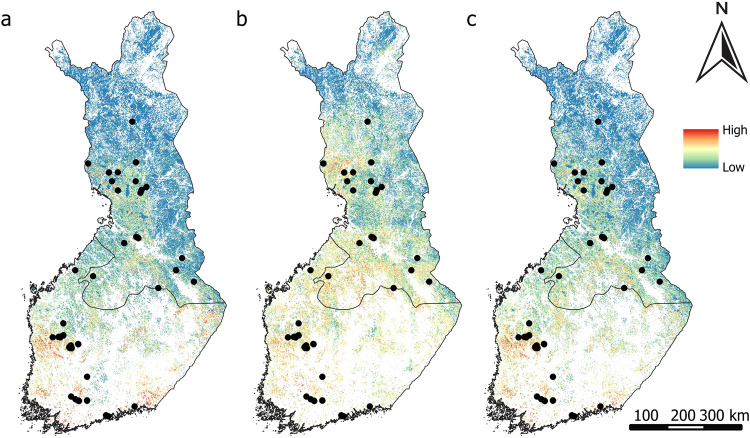
Predicted probability of CH_4_ sources from (**a**) geospatial environmental variables, (**b**) remote sensing variables, (**c**) both geospatial environmental and remote sensing variables. Black dots represent the measurement sites of CH_4_ sources

CH_4_ sources were mainly predicted in Finland’s western, middle, and southern regions according to the geospatial environmental variables (Fig. 5a), and when both geospatial environmental and remote sensing variables were used (Fig. 5c). Remote sensing data extended these predictions to include the northern area as well (Fig. 5b).

**Fig. 6:**
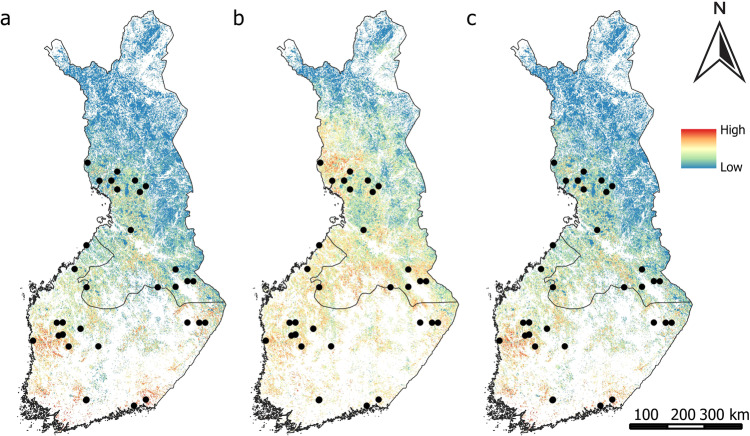
Predicted probability of CO_2_ sinks from (**a**) geospatial environmental variables, (**b**) remote sensing variables, (**c**) geospatial environmental and remote sensing variables. Black dots represent the field measurement sites of CO_2_ sinks

CO_2_ sinks were primarily predicted to be concentrated in the western, middle, and southern parts of the country according to the geospatial environmental variables (Fig. 6a) and when both geospatial environmental and remote sensing variables were used (Fig. 6c), while they were predicted also for northwestern, and northeastern parts when using solely remote sensing data (Fig. 6b).

**Fig. 7:**
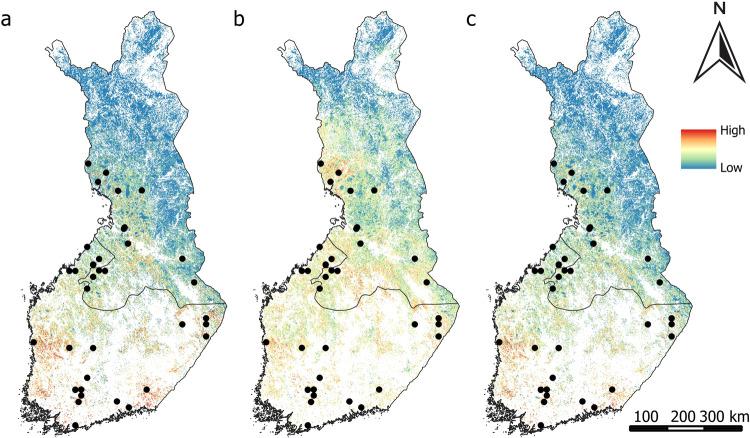
Predicted probability of CO_2_ sources from (**a**) geospatial environmental variables, (**b**) remote sensing variables, (**c**) geospatial environmental and remote sensing variables. Black dots represent the field measurement sites of CO_2_ sources

Environmental variables predicted CO_2_ sources predominantly in Finland’s western, middle to southern regions (Fig. 7a) and also when both geospatial environmental and remote sensing variables were used (Fig. 7c). Remote sensing data extended these predictions to include the northern area as well (Fig. 7b).

**Fig. 8:**
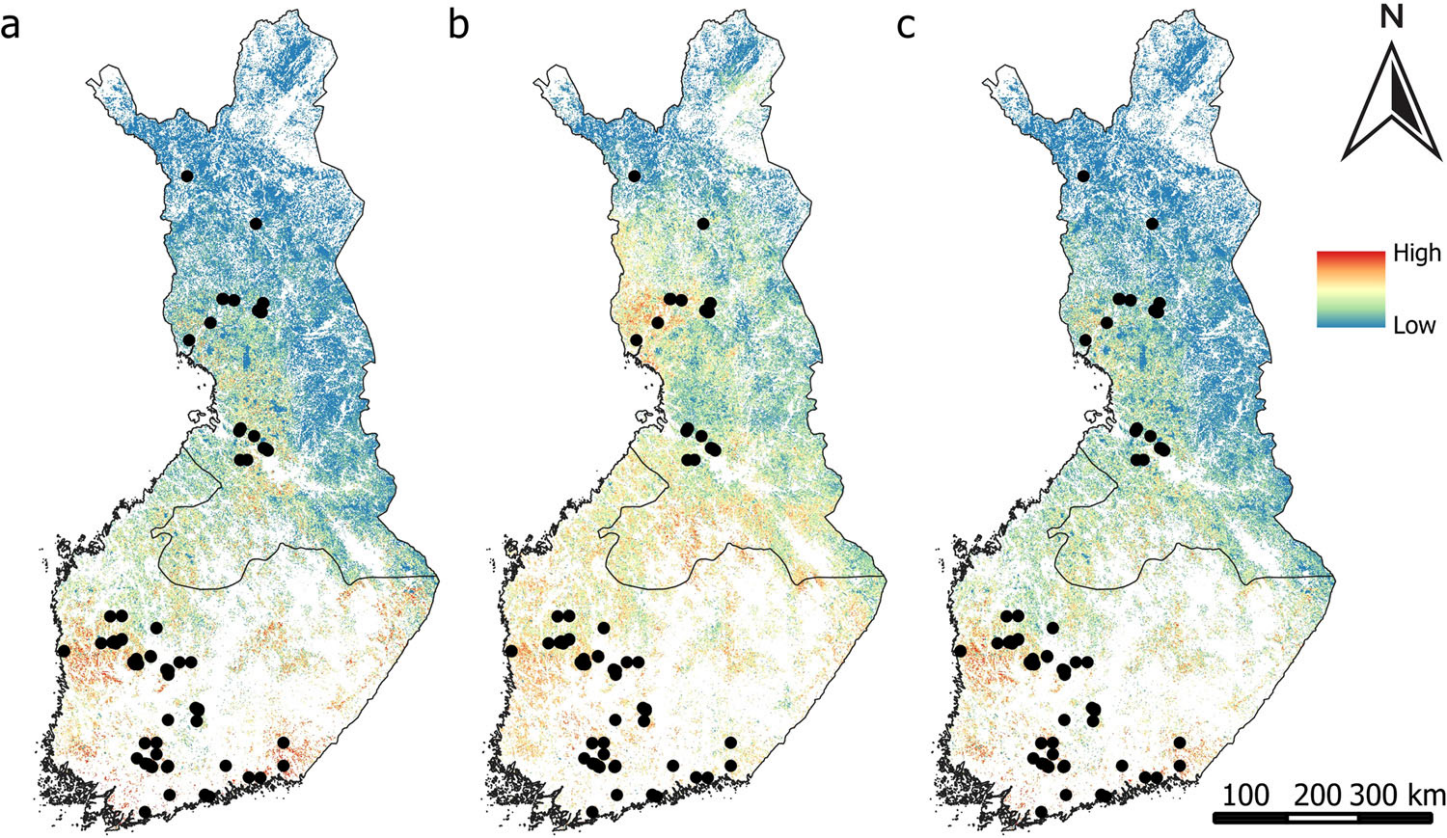
Predicted probability of N_2_O sources from (**a**) geospatial environmental variables, (**b**) remote sensing variables, (**c**) geospatial environmental and remote sensing variables. Black dots represent the measurement sites of N_2_O sources

NO_2_ sources were primarily concentrated in the western, middle to southern parts of the country according to the geospatial environmental variables (Fig. 8a) and when integrating both geospatial environmental and remote sensing variables (Fig. 8c). Remote sensing variables depicted a more dispersed distribution, extending from the northern to southern regions of the country (Fig. 8b).

## Discussion

### GHG model accuracies

Our study shows that the spatial distribution of GHG sinks and sources on a national scale can be predicted using either a combination of geospatial environmental and remote sensing data or solely geospatial environmental data. The predictive accuracy and stability remained consistent across all models, indicating their robustness for spatial prediction. Variables reflecting drainage intensity and climate consistently performed well in all GHG models, highlighting their significant influence as the primary drivers of GHG sinks and sources.

Models relying solely on remote sensing variables demonstrated lower predictive accuracy than the two other model types. This suggests that using only remote sensing data is not optimal for predicting GHG sinks and sources over large spatial extents. Nonetheless, integrating remote sensing data with environmental GIS data slightly improves model accuracy. This highlights the importance of incorporating land cover, vegetation, and moisture-related proxies from remote sensing data to better understand the spatial patterns of GHG sinks and sources. This result concurs with earlier studies which emphasize that multiple different data sources should be used when producing maps for biogeochemical and ecological phenomena such as GHG fluxes, vegetation, and land cover (Karlson et al. 2019; Räsänen et al. 2021; Räsänen and Virtanen 2019; White et al. 2017).

### Spatial patterns of the prediction maps

The GHG distribution map derived from remote sensing variables displayed a slightly different spatial pattern compared to the maps generated using geospatial environmental variables alone or in combination with both types of variables. These disparities can be attributed to the inherent differences in the nature of the data sources utilized. Geospatial environmental data quantify variables such as drainage intensity, habitat type, topography, and climate, which are closely linked to GHG fluxes between ecosystems and the atmosphere. In contrast, satellite data rely on detecting surface properties (e.g., vegetation type, land cover) and physical phenomena (e.g., soil moisture, temperature) that can indirectly influence GHG emissions. However, these relationships may not always be straightforward or consistent across different regions and ecosystems, leading to uncertainties in the predictive models.

Generally, the prediction maps identified a higher probability of GHG sources towards the southern area. One reason may be the overall increase in drainage towards the south, coupled with more intensive degradation of peatlands in that region. In addition, GHG sinks were also often predicted to occur in the same grid cells as GHG sources. This might be caused by the spatial heterogeneity of land use and land cover within the region. While certain areas experience extensive peatland degradation and subsequent GHG emissions due to drainage and land conversion activities, other nearby areas may retain relatively intact vegetation. The juxtaposition of these contrasting land cover and management types within the same grid cells can result in the coexistence of GHG sinks and sources. Additionally, the complex interplay of factors such as soil properties, hydrological dynamics, and management practices further contributes to the variability in GHG fluxes observed at the local scale (Abdalla et al. 2016; Bhullar et al. 2013; Koch et al. 2023). For instance, the different GHGs respond differently to drainage and management activities, with pristine peatlands being predominantly CO_2_ sinks and CH_4_ sources, while forestry-drained peatlands are typically CO_2_ sources (Joosten and Clarke 2002; Kaat and Joosten 2009; Pönisch et al. 2023). Consequently, despite the prevalence of GHG sources in the southern area, the presence of GHG sinks within the same grid cells highlights the importance of considering the multifaceted nature of landscape processes in predicting regional GHG dynamics.

### Geospatial environmental variables influencing GHG

Our findings showed that UNDRAINED, DRAINED, and GDD were the most significant geospatial environmental variables in explaining the GHG sink and source distributions, which corroborates with the study by Parkkari et al. (2017). UNDRAINED and DRAINED, habitat-related variables, represents the proportion of undrained and drained peatlands. This variable serves as an important explanatory factor for GHG sinks and sources due to the fact that the presence of drainage significantly alters peatland hydrology and biogeochemical processes related to GHGs (Hyvönen et al. 2013; Laine et al. 2019; Laine et al. 1996).

Climate variables such as GDD and WAB were important in explaining the spatial patterns of CH_4_ and N_2_O. It is somewhat surprising that, in our models, these climate variables had a higher significance for CH_4_ and N_2_O compared to CO_2_, suggesting that the influence of climate variables on CO_2_ was overshadowed by the higher significance of other variables. It is probably because drained peatlands undergo substantial alterations in terms of water table levels, soil conditions, and vegetation types, which are more directly linked to CO_2_ release. Site type and fertility further influence the availability of organic matter and nutrient cycling, directly impacting CO_2_ emissions. While climatic variables play a role, the local peatland characteristics have a more immediate and profound impact on the CO_2_ dynamics, making them more dominant factors in the predictive model. Particularly, GDD has an impact on CO_2_ balance since the length of the growing season increases photosynthesis activity and thus CO_2_ uptake (Gatis et al. 2019; Zhu et al. 2022). However, it may be that GDD primarily affects the strength of CO_2_ uptake rather than determining whether a specific area acts as a net sink or source of CO_2_ (Groendahl et al. 2007; Kroner and Way 2016). Other factors, such as organic matter decomposition and soil moisture, might play more significant roles in dictating the overall CO_2_ balance (Castro et al. 2010; Clark et al. 2009; Cregger et al. 2014; Wilson et al. 2022).

Additionally, some other habitat variables, representing site fertility information, were deemed important in many of the GHG models. For example, unfertile or nutrient-poor site (jätkg) influenced the CH_4_ sink model and moderately fertile site (mtkg) and less fertile site (ptkg) the CO_2_ sink model. This observed relationship can be attributed to the impact of nutrient availability on ecosystem functioning. In moderately fertile or less fertile sites, microbial activity driven by organic matter decomposition may be enhanced under nutrient-limited conditions (Bhullar et al. 2013; Koelbener et al. 2010), leading to elevated methane emissions and influencing the CH_4_ sink/source dynamics. Limited nutrient availability may also constrain plant productivity and carbon sequestration potential, resulting in reduced CO_2_ sink strength (Hommeltenberg et al. 2014; Lohila et al. 2011; Ojanen et al. 2013). On the other hand, fertile sites might release more carbon into the atmosphere than they capture due to their high respiration and productivity levels, contributing to the climate warming (Jauhiainen et al. 2016; Maljanen et al. 2010; Ojanen et al. 2013; Renou-Wilson et al. 2014). Furthermore, variations in vegetation composition and litter decomposition rates associated with nutrient availability further contribute to the observed patterns in GHG fluxes. The relationship between peatland site fertility and GHG sinks and sources is interconnected with other factors, such as water table depth, temperature, vegetation composition, and land management practices (e.g., drainage and fertilization) (Kareksela et al. 2015; Laine et al. 2019; Soini et al. 2010).

The contribution of TWI was minimal in this study, possibly due to ditching, which likely alters the hydrological characteristics of the landscape and may have a significant impact on soil moisture dynamics, overriding the influence of TWI (Parkkari et al. 2017).

### Remote sensing variables influencing GHG

Our results highlight the importance of considering multitemporal remote sensing variables derived from different stages of the growing season when predicting GHG dynamics. By examining data from early summer, mid-summer, and late summer, we captured variations in vegetation growth and temporal moisture conditions that influence GHG sinks and sources.

The results showed that Sentinel-2 data had higher predictive power compared to Sentinel-1 data, likely due to the effectiveness of optical data in detecting peatland wetness, especially in open peatlands and areas where wetness correlates with land cover and vegetation patterns (Burdun et al. 2020; Räsänen et al. 2020). Sentinel-2 variables were also ranked in the top three most influential variables, even when considered alongside environmental variables. This suggests that incorporating Sentinel-2 data has the potential to improve the accuracy and reliability of GHG sinks and sources predictions.

Across various GHG models, most bands and indices derived from Sentinel-2 data consistently ranked within the top three, except for the green and SWIR bands. The low importance of SWIR is a bit surprising as SWIR bands have been identified as sensitive indicators of moisture content, both in vegetation (Ceccato et al. 2001) and soil (Crist and Cicone 1984) and also important in predicting restored and intact peatland water table depths (Burdun et al. 2023; Räsänen et al. 2022). Individual bands such as BLUE, RED, RE1, RE2, and NIR emerged as the most important ones. These bands have been previously identified as useful in estimating soil moisture and vegetation cover (Junttila et al. 2021; Kolari et al. 2022; Pang et al. 2023). Moreover, our study also identified moisture and vegetation indices as important variables in predicting GHG sinks and sources. These indices provide valuable information about surface soil moisture content, water presence, and vegetation density (Lees et al. 2020; Räsänen et al. 2022). However, it is essential to note that the effectiveness of optical data, such as Sentinel-2, to detect soil moisture, ground vegetation, and land cover diminishes in peatlands densely covered by trees (Burdun et al. 2023; Räsänen et al. 2022) due to the obstructive nature of the tree canopy.

Despite the superior performance of Sentinel-2, it is noteworthy that Sentinel-1 variables also held an important role in our GHG models. VH (Vertical-Horizontal) variables from Sentinel-1 were ranked among the top three most important variables in CO_2_ sinks model. Earlier studies have emphasized the sensitivity of Sentinel-1 and other SAR data to soil moisture, proving valuable in mapping peatland vegetation, land cover, moisture and GHG fluxes (Bourgeau-Chavez et al. 2009; Karlson et al. 2019; Millard et al. 2020; Räsänen et al. 2021; White et al. 2017). However, as a C-band satellite, Sentinel-1 may not be optimal for moisture mapping due to its limited penetration capabilities through vegetation. The notable contribution of Sentinel-1 variables, even when compared to Sentinel-2, underscores the complementary role of these two remote sensing datasets. This highlights the significance of leveraging multiple remote sensing datasets for a comprehensive understanding and modeling of GHG dynamics in peatland ecosystems.

### Limitations and future directions

There were some limitations in our study which should be addressed in future studies. Firstly, our GHG data were measured from a limited number of sites, exclusively focusing on several years, with data collected mostly during the snow and frost-free season, which were then used to estimate the annual GHG balance. This restricted spatial and temporal coverage may hinder the comprehensive capture of fluctuations in GHG sinks and sources across different seasons and geographical locations. To address this limitation in future investigations, expanding the field dataset to include a broader range of sites, covering various seasons, could provide a more nuanced understanding of peatland GHG dynamics.

Secondly, there is a bias towards drained peatland sites in our study, with limited representation of undrained and rewetted sites. This bias may affect the generalizability of our findings, especially concerning GHG dynamics in undrained and rewetted peatlands. To improve the overall understanding of GHG dynamics in peatland ecosystems, future studies should aim for a more balanced dataset that includes undrained and rewetted sites.

Thirdly, while our study successfully identified spatial patterns of GHG sinks and sources, it did not explore the strength of these sinks and sources. This limitation restricts the depth of understanding of peatland GHG dynamics. Future work should delve into quantifying the strength of GHG sinks and sources to provide a more comprehensive understanding of their impact on the overall carbon balance in peatland ecosystems.

After all, a GHG predictive model is essential to identify areas with high GHG emissions or sequestration potential. Such information holds significant value for land-use planning, empowering decision-makers to allocate resources effectively and prioritize areas for conservation, restoration, or economic use. The integration of predictive models into decision making processes can contribute to more informed and environmentally conscious land-use practices. However, the maps should not be used directly to prioritize areas in spatial decision making. Instead, the results should be validated and discussed together with decisionmakers and other stakeholders (Hauck et al. 2013). Such discussion can be even more important than the result maps themselves as the discussions facilitate social learning and knowledge exchange between various sectors and help to understand the environmental processes relevant for GHG dynamics. Nevertheless, as the maps provide easily comprehensive and illustrative information, they are important for facilitating such discussions. Therefore, further research could look at how the maps can be used in decision making.

## Conclusion

Our study demonstrates that the combination of geospatial environmental and remote sensing data can predict peatland GHG sinks and sources on a large spatial extent. Geospatial environmental variables like drainage and climate-related variables were the most important contributors to the models. Models relying solely on remote sensing variables from Sentinel-1 and Sentinel-2 performed worse than those using geospatial environmental variables. However, the combination of remote sensing and geospatial environmental variables slightly boosted model performance compared to models utilizing only geospatial environmental variables. The maps generated from environmental variables alone and those from the combined dataset display similarity, indicating the robustness of the approach. Nonetheless, maps based solely on remote sensing data showed slightly different patterns. These results suggest that (1) reliable nationwide estimates of GHG sinks and sources cannot be produced with remote sensing data only and (2) integrating multiple data sources is recommended to achieve accurate and realistic predictions of GHG spatial patterns in peatland ecosystems.

### Supplementary information

Supplementary Information

## Data Availability

The data and code utilized in this study are available upon request from the corresponding author.
