# Supplementary material for: Detecting Spatial Patterns of Peatland Greenhouse Gas Sinks and Sources with Geospatial Environmental and Remote Sensing Data
Source: Environ Manage. 2024 Apr 2;74(3):461–78. doi: 10.1007/s00267-024-01965-7 (PMC11306394; doi:10.1007/s00267-024-01965-7)
Supplement: Supplementary file 1 — Supplementary Information [file 267_2024_1965_MOESM1_ESM.docx]

**Supplementary information**

Table S1. Important variables contributing to the CH_4_ sink model.

| Env | Permutation importance | RS | Permutation importance | Env & RS | Permutation importance |
| --- | --- | --- | --- | --- | --- |
| GDD | 35.395 | ES_Blue | 18.793 | undrained | 23.162 |
| undrained | 27.646 | MS_RE1 | 18.753 | GDD | 17.707 |
| jatkg | 15.761 | MS_NDWI | 16.461 | MS_NDWI | 14.872 |
| drained | 11.991 | MS_RE2 | 15.858 | jatkg | 8.297 |
| mtkg | 5.701 | ES_NDWI | 9.319 | drained | 8.000 |
| rhtkg | 3.507 | MS_Blue | 7.120 | MS_RE2 | 6.942 |
|  |  | ES_POL | 3.497 | mtkg | 5.037 |
|  |  | ES_NDMI | 3.096 | ES_Blue | 4.768 |
|  |  | ES_SWIR1 | 2.722 | ptkg | 4.179 |
|  |  | MS_VH | 2.260 | ES_POL | 3.794 |
|  |  | MS_NDMI | 2.121 | MS_NDVI | 3.243 |

Table S2. Important variables contributing to the CH_4_ source model.

| Env | Permutation importance | RS | Permutation importance | Env & RS | Permutation importance |
| --- | --- | --- | --- | --- | --- |
| drained | 30.556 | MS_NDMI | 17.920 | ES_MNDWI | 13.368 |
| GDD | 18.838 | ES_RED | 13.772 | GDD | 11.681 |
| WAB | 17.154 | MS_NDVI | 13.458 | MS_NDVI | 11.056 |
| mtkg | 13.262 | ES_MNDWI | 11.039 | drained | 9.848 |
| jatkg | 8.584 | LS_VH | 9.350 | ES_RE2 | 8.894 |
| ptkg | 5.088 | ES_VV | 8.459 | ES_RED | 6.581 |
| rhtkg | 3.463 | ES_VH | 7.885 | WAB | 5.146 |
| undrained | 3.055 | MS_POL | 7.048 | ES_VV | 4.925 |
|  |  | ES_RE2 | 5.248 | MS_NDMI | 4.651 |
|  |  | MS_Blue | 2.383 | ES_NDMI | 4.606 |
|  |  | ES_NDWI | 2.009 | MS_SWIR1 | 4.261 |
|  |  | ES_POL | 1.430 | MS_SWIR2 | 4.005 |
|  |  |  |  | LS_VH | 3.079 |
|  |  |  |  | mtkg | 2.702 |
|  |  |  |  | jatkg | 2.082 |
|  |  |  |  | ES_VH | 1.234 |
|  |  |  |  | undrained | 0.703 |
|  |  |  |  | MS_POL | 0.637 |
|  |  |  |  | TWI | 0.541 |

Table S3. Important variables contributing to the CO_2_ sink model.

| Env | Permutation importance | RS | Permutation importance | Env & RS | Permutation importance |
| --- | --- | --- | --- | --- | --- |
| mtkg | 19.095 | LS_VH | 63.547 | undrained | 15.372 |
| undrained | 17.389 | MS_Blue | 15.087 | GDD | 12.331 |
| ptkg | 17.094 | ES_Blue | 9.486 | drained | 10.945 |
| drained | 16.124 | ES_POL | 6.335 | ES_VV | 10.643 |
| GDD | 13.807 | ES_NIR | 5.545 | mtkg | 10.400 |
| WAB | 5.975 |  |  | ES_NIR | 7.669 |
| TWI | 5.578 |  |  | ptkg | 7.608 |
| rhtkg | 4.937 |  |  | MS_SWIR2 | 6.334 |
|  |  |  |  | MS_VH | 5.655 |
|  |  |  |  | WAB | 5.567 |
|  |  |  |  | ES_VH | 2.763 |
|  |  |  |  | TWI | 2.646 |
|  |  |  |  | MS_NDWI | 2.067 |

Table S4. Important variables contributing to the CO_2_ source model.

| Env | Permutation importance | RS | Permutation importance | Env & RS | Permutation importance |
| --- | --- | --- | --- | --- | --- |
| undrained | 35.425 | MS_RE1 | 33.509 | MS_RE1 | 18.083 |
| drained | 27.309 | ES_NIR | 18.890 | undrained | 17.146 |
| GDD | 14.747 | ES_RE2 | 15.542 | GDD | 14.142 |
| mtkg | 9.454 | MS_NDVI | 10.078 | drained | 12.265 |
| jatkg | 7.085 | MS_Blue | 7.774 | ES_NDMI | 10.922 |
| ptkg | 3.939 | ES_POL | 3.303 | MS_NDVI | 8.733 |
| WAB | 2.041 | MS_MNDWI | 2.637 | MS_POL | 4.921 |
|  |  | MS_VH | 2.550 | jatkg | 3.911 |
|  |  | ES_NDMI | 2.187 | MS_Blue | 3.827 |
|  |  | ES_Blue | 1.828 | MS_NDWI | 3.411 |
|  |  | MS_NDWI | 1.702 | mtkg | 1.376 |
|  |  |  |  | MS_VH | 1.264 |

Table S5. Important variables contributing to the N_2_O source model.

| Env | Permutation importance | RS | Permutation importance | Env & RS | Permutation importance |
| --- | --- | --- | --- | --- | --- |
| GDD | 28.753 | ES_MNDWI | 17.812 | WAB | 17.017 |
| WAB | 27.255 | ES_RED | 11.752 | GDD | 16.481 |
| drained | 19.787 | MS_MNDWI | 8.448 | ES_NIR | 15.762 |
| jatkg | 8.859 | MS_NDVI | 7.721 | drained | 11.986 |
| undrained | 6.947 | ES_NDVI | 7.308 | ES_MNDWI | 8.323 |
| rhtkg | 3.190 | ES_POL | 6.375 | ES_RED | 7.506 |
| ptkg | 3.161 | ES_Blue | 6.178 | jatkg | 6.997 |
| mtkg | 2.048 | LS_VH | 5.794 | ES_Blue | 6.821 |
|  |  | ES_RE2 | 4.448 | rhtkg | 3.753 |
|  |  | ES_VH | 4.215 | MS_NDVI | 3.159 |
|  |  | MS_RE1 | 2.812 | ES_POL | 2.070 |
|  |  | MS_Blue | 2.611 | ES_RE2 | 0.125 |
|  |  | ES_NIR | 2.432 |  |  |
|  |  | MS_NIR | 2.363 |  |  |
|  |  | ES_RE1 | 2.069 |  |  |
|  |  | MS_RED | 2.058 |  |  |
|  |  | ES_VV | 1.639 |  |  |
|  |  | MS_VV | 1.358 |  |  |
|  |  | ES_NDMI | 1.346 |  |  |
|  |  | MS_POL | 1.262 |  |  |
